# Association of a platelet function testing-guided intensified antiplatelet strategy with thromboembolic events after EP2 stent-assisted coil embolization in patients with unruptured intracranial aneurysms

**DOI:** 10.3389/fneur.2026.1829631

**Published:** 2026-09-22

**Authors:** Jinan Bai, Xin Li, Ailiang Ge, Shibo Sun, Changjia Yang, Chen Liang, Yong Zhen

**Affiliations:** 1 Neurosurgery Department of Yangzhou Hongquan Hospital, Yangzhou, China; 2 Department of Neurosurgery, Northern Jiangsu People’s Hospital Afffliated to Yangzhou University, Yangzhou, China

**Keywords:** dual antiplatelet therapy, high on-treatment platelet reactivity, platelet function testing, tirofiban, unruptured intracranial aneurysms

## Abstract

**Introduction:**

To evaluate the association of Platelet Function Testing (PFT)-guided intensified antiplatelet strategy with the incidence of thromboembolic events and bleeding rates in patients with Unruptured Intracranial Aneurysms (UIA) undergoing stent-assisted coil embolization, compared with standard fixed-dose Dual Antiplatelet Therapy (DAPT).

**Methods:**

In this retrospective study, 368 patients from two centers between January 2019 and September 2024 were enrolled and categorized into a Monitored Group (*n* = 219) and a Non-monitored Group (*n* = 149). Both groups initially received a fixed-dose DAPT regimen of clopidogrel 75 mg and aspirin 100 mg daily. Within 24 h prior to the procedure, the Monitored Group underwent PFT. Patients exhibiting High On-Treatment Platelet Reactivity (HPR) to clopidogrel were administered a single 300 mg clopidogrel loading dose and received short-term perioperative tirofiban. For patients with HPR to aspirin, the aspirin dose was increased to 200 mg daily. The Non-monitored Group did not undergo platelet function testing and dose adjustment. To control for confounding, propensity score matching (PSM) and multivariable Cox regression were performed. The primary outcome was the composite thromboembolic event rate within 7 days post-procedure. Secondary outcomes included the composite thromboembolic event rate within 30 days, and the safety outcome was the composite bleeding event rate within 30 days.

**Results:**

In the original cohort, the monitored strategy was associated with a lower incidence of the primary 7-day composite thromboembolic outcome (3.20% vs. 8.05%; unadjusted HR = 0.388, 95%CI 0.153–0.985, *p* = 0.045; adjusted HR = 0.391, 95%CI 0.154–0.994, *p* = 0.048). The association remained directionally consistent in the propensity score-matched cohort (115 pairs, 4.35% vs. 8.70%; HR = 0.487, 95%CI 0.166–1.424, *p* = 0.189). The secondary 30-day composite thromboembolic outcome showed similar patterns (original cohort: adjusted HR = 0.412, 95%CI 0.187–0.909, *p* = 0.028; PSM cohort: HR = 0.446, 95%CI 0.169–1.172, *p* = 0.101). No statistically significant association was observed for the 30-day composite bleeding event rates between the two groups in either the original or matched analyses. Subgroup analyses identified no significant association modifiers.

**Conclusion:**

A PFT-guided intensified antiplatelet strategy was associated with a lower incidence of early post-procedural thromboembolic events without a significant increase in major bleeding in UIA patients treated with EP2 stent-assisted coiling. These findings support the potential utility of this comprehensive strategy, although the independent contribution of each component requires further investigation in larger prospective studies.

## Introduction

Endovascular neurointerventional therapy is currently the primary and most frequently employed method for treating intracranial aneurysms (1). This approach encompasses techniques such as coil embolization, stent-assisted coiling, and flow diversion (2, 3). Among these, stent-assisted coil embolization, particularly utilizing the Enterprise 2 (EP2) stent, has become a pivotal modality for managing Unruptured Intracranial Aneurysms (UIAs) (4). This technique enhances coil stability, improves the immediate occlusion rate of the aneurysm, and, owing to the stent’s superior conformability and geometric design, holds promise for reducing long-term recanalization risks. Furthermore, the EP2 stent, characterized by a larger cell size and improved wall apposition compared to earlier generations, facilitates dense aneurysm packing and favorable outcomes (5). However, peri-procedural thromboembolic events (TEEs) represent a major complication impacting patient functional prognosis. Epidemiological studies indicate a peri-procedural cerebral infarction rate of approximately 5.7%, with a permanent complication rate of about 1.9%, figures comparable to other stent-assisted coiling series (5). Incomplete stent apposition, particularly in tortuous vessels, can potentiate thrombus formation, potentially leading to disabling neurological deficits (6).

The current standard of care involves peri-procedural fixed-dose Dual Antiplatelet Therapy (DAPT), typically oral aspirin 100 mg and clopidogrel 75 mg once daily, as recommended by current guidelines (7). However, significant interindividual variability in drug response exists, and some patients exhibit High On-Treatment Platelet Reactivity (HPR), defined as insufficient platelet inhibition despite regular DAPT, which is associated with a heightened risk of thrombotic events (8). HPR has been reported in approximately 30–50% of Asian patients undergoing neurointerventional procedures, primarily due to a high prevalence of CYP2C19 loss-of-function alleles (9–12). Platelet Function Testing (PFT) has been proposed to identify patients with HPR and guide antiplatelet therapy adjustment (13). Several observational studies have suggested that PFT-guided adjustment may be associated with a reduced risk of thromboembolic complications (13, 14). However, the optimal peri-procedural adjustment strategy including PFT-based screening, loading dose, aspirin dose escalation, and short-term tirofiban bridging in patients identified as HPR—remains incompletely evaluated in the context of EP2 stent-assisted coiling. Tirofiban, as an intravenous glycoprotein IIb/IIIa receptor antagonist with rapid onset and short half-life, has been used as a bridging agent during the high-risk peri-procedural period, but its role as part of a PFT-guided strategy requires further investigation (15).

Despite the theoretical benefits and increasing clinical adoption of the PFT-guided individualized strategy, its association with clinical outcomes and its safety profile specifically in the context of EP2 stent-assisted coiling, particularly when combined with tirofiban bridging therapy, still lack systematic evaluation. Therefore, the purpose of this study was to assess the association of a PFT-guided intensified antiplatelet strategy with the incidence of thromboembolic events, compared with standard fixed-dose DAPT in UIA patients undergoing EP2 stent-assisted coil embolization.

## Materials and methods

### Case selection

A retrospective screening was conducted on 388 patients with UIA who underwent EP2 stent-assisted coiling between January 2019 and September 2024 at the neurosurgical centers of Yangzhou Hongquan Hospital and Northern Jiangsu People’s Hospital. Patients were allocated to the two groups based on calendar period and institutional availability rather than on patient characteristics or operator preference. Specifically, patients treated before 2022 did not undergo PFT because it was not yet routinely available at either center (Non-monitored Group, *n* = 149), whereas those treated from 2022 onward received PFT as part of standard pre-procedural assessment (Monitored Group, *n* = 219). Ultimately, 368 patients were included in the analysis (Figure 1). This study was approved by the Ethics Committees of Yangzhou Hongquan Hospital and Northern Jiangsu People’s Hospital (Approval Nos.: 2025–015; 2017KY-090). All data were anonymized to preserve patient privacy, and all study practices closely followed the guidelines set forth by the relevant clinical ethics committee as well as the ethical standards outlined in the Declaration of Helsinki of the World Medical Association. The sample size was estimated based on the event rates observed in our preliminary data. Assuming a thromboembolic event rate of 8.0% in the non-monitored group and 3.0% in the monitored group [based on the observed incidence in the present cohort and consistent with previously reported rates of 6.3–9.4% for stent-assisted coiling in unruptured aneurysms (5)], a sample size of 105 patients per group was estimated to provide 80% power at a two-sided significance level of 0.05 to detect a difference in the primary outcome.

**Figure 1:**
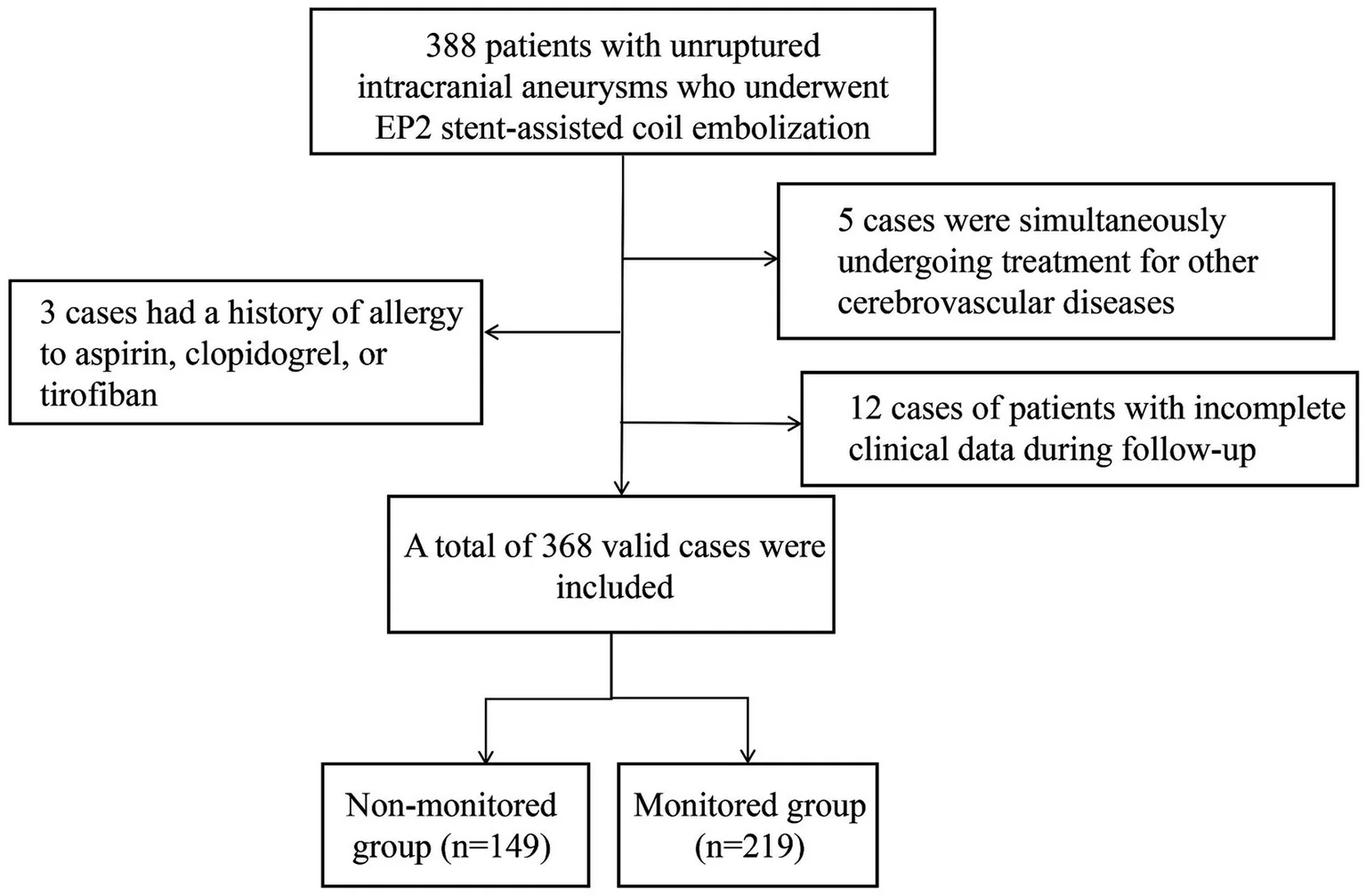
Study flow diagram. EP2, Enterprise 2.

Diagnostic Criteria: Referenced the *Chinese guideline for the clinical management of patients with unruptured intracranial aneurysms (2024)* (16). Inclusion Criteria: (1) Aged between 18 and 75 years; (2) Met the aforementioned diagnostic criteria; (3) Diagnosed with a UIA and scheduled for stent-assisted coiling; (4) Had a modified Rankin Scale (mRS) score of less than 2 on the day of enrollment. Exclusion Criteria: (1) Experienced complications related to the surgical procedure or device, such as guidewire perforation of a vessel, coil herniation, or incompletely opened stents; (2) were being treated for additional cerebrovascular conditions at the same time; (3) had a documented history of tirofiban, clopidogrel, or aspirin allergies; (4) Were pregnant or lactating women; (5) Had severe hepatic or renal insufficiency, malignancy, or immunodeficiency; (6) Had previously undergone endovascular stent implantation for intracranial aneurysm treatment; (7) had a high risk of coagulopathy or active bleeding (such as symptomatic cerebral hemorrhage or an active stomach ulcer); (8) were taking anticoagulants; (9) were either lost to follow-up or had insufficient medical data. General Information: The hospital’s electronic medical record system provided all of the data used in this investigation. To ensure data quality, a unified data extraction protocol was employed. Two researchers independently reviewed the medical records, and any disputed data were adjudicated by a third senior physician.

### Preprocedural platelet function testing

The blood sample collection procedure for all patients followed a unified standard. According to medical records, patients were in a resting state prior to phlebotomy, had abstained from smoking for at least 30 min and from caffeine for more than 2 h, and no history of high-fat diet intake was reported. Blood sampling was performed using a 21-gauge needle from the antecubital fossa vein and was consistently scheduled within 2 to 4 h after the administration of antiplatelet medication. To minimize platelet activation, the initially drawn 2–4 mL of blood was routinely discarded, and all samples were processed within 1 h of collection. Light transmission aggregometry (LTA) and flow cytometry were used to evaluate platelet function. Arachidonic acid (AA) and adenosine diphosphate (ADP) were employed as agonists to assess the patient’s reaction to aspirin and clopidogrel, respectively (17, 18). In order to extract platelet-rich plasma (PRP), citrated anti-coagulated whole blood was centrifuged at 800 rpm for 10 mins. To get platelet-poor plasma (PPP), the residual blood fraction was then centrifuged again for 10 mins at 2500 rpm. For the assay, samples were stimulated with 5 μmol/L ADP and 1 mmol/L AA, respectively, and platelet aggregation rates were measured using a light transmission aggregometer (Model AG800, Beijing Taimeng Biotechnology Co., Ltd.).

### Antiplatelet therapy

Based on the antiplatelet therapy regimens actually received by patients under the guidance of the *Chinese Expert Consensus on Antiplatelet Therapy for Intracranial Aneurysms* (7), every patient was split into two groups: (1) Non-monitored Group (antiplatelet medication at a fixed dose without platelet reactivity monitoring): Five days before to the procedure, antiplatelet treatment consisting of 75 mg of clopidogrel and 100 mg of aspirin was started once daily. (2) Monitored Group (individualized antiplatelet therapy guided by PFT): Based on the regimen of the Non-monitored Group, PFT was performed 1 day before the procedure. In patients who showed HPR to clopidogrel, the 75 mg clopidogrel dose was substituted with a single 300 mg clopidogrel loading dose before to surgery, along with intraoperative and short-term postoperative usage of tirofiban. Tirofiban was administered as an intravenous bolus of 10 μg/kg followed by a continuous infusion of 0.15 μg/kg/min for 12 h postoperatively. The infusion was discontinued 12 h after initiation, overlapping with oral DAPT for at least 4 h to ensure a smooth transition. The aspirin dosage was raised to 200 mg once daily for patients who showed HPR to the medication. The initial regimen was followed if patients showed a sufficient response or low platelet reactivity to the dual antiplatelet medication of 75 mg clopidogrel and 100 mg aspirin (all dose modifications were conducted within 24 h prior to the surgery). Stent implantation in the Monitored Group was completed within 24 h after medication adjustment. Aspirin 100 mg once daily and clopidogrel 75 mg once daily were administered to all patients in both groups as long-term postoperative medication, which was stopped 3 months after surgery. HPR was defined as a maximum platelet aggregation (MPA) of ≥42.9% induced by ADP (19), and insufficient response to aspirin was defined as an MPA of ≥20% induced by AA (20).

### Routine protocol for stenting treatment of patients with unruptured intracranial aneurysm

The patient was positioned supine in the catheterization laboratory. The surgical site was regularly cleaned with iodophor and covered with sterile cloths following the induction of general anesthesia. The femoral artery was punctured using the Seldinger technique approximately 0.5 cm below the inguinal ligament at the point of maximum pulsation, and an 8F introducer sheath (Terumo Corporation, Japan) was successfully placed. A 5F diagnostic catheter was advanced into both vertebral arteries, and angiography confirmed the presence of a basilar tip aneurysm. A 6F Envoy guiding catheter (Cordis, USA) was inserted into the vertebral artery using a 0.035-inch guidewire (Terumo Corporation, Japan) following the removal of the diagnostic catheter. A plan of action was created. A Synchro-select microguidewire (Stryker, USA) was then used to insert a Prowler Select Plus microcatheter (Codman & Shurtleff, Inc., USA) into the P2 segment of the posterior cerebral artery. Guided by a neuro microguidewire (GZ Easy Guide, China), a Headway-17 microcatheter (MicroVention, USA) was navigated into the basilar artery and carefully introduced into the aneurysm sac. Coils (ev3, USA) were deployed into the aneurysm. Due to a wide neck, the coils were unstable. An Enterprise 2 stent (Codman, USA) was deployed to cover the aneurysm neck. The coils were subsequently repositioned, achieving stability, and were then detached. The aforementioned steps were repeated, sequentially deploying a 2 mm × 6 cm Axium Prime coil and a 1.5 mm × 2 cm Axium Prime coil (ev3, USA). Control angiography demonstrated complete occlusion of the aneurysm with patency of the surrounding vessels. The procedure was terminated. All microcatheters and the guide catheter were withdrawn. The femoral artery access site was closed using a vascular closure device (Abbott, USA). The patient was awakened from general anesthesia, extubated, and transferred back to the ward.

### Imaging protocols

Endovascular interventional procedures were performed using biplane digital subtraction angiography systems equipped with three-dimensional rotational angiography functionality (models from Siemens Healthineers, Philips Healthcare, and GE Healthcare). To rule out intracranial hemorrhage, subjects exhibiting neurological impairments first received non-contrast computed tomography (CT) of the head. A 256-slice CT scanner (GE Healthcare Revolution CT) operating in axial acquisition mode was used for these scans. Magnetic resonance imaging (MRI) was then carried out utilizing a 3 Tesla MRI system (Siemens Healthineers Magnetom Prisma) with a 64-channel head/neck coil if no bleeding was found. All imaging data were evaluated by an independent imaging core laboratory, whose assessors were blinded to the treatment group assignment (Monitored Group vs. Non-monitored Group). Two neurointerventional physicians independently reviewed the images; any discrepancies were resolved by a senior radiologist whose decision was final. A Clinical Events Committee, whose members were also blind to the treatment allocation, independently evaluated each endpoint occurrence.

### Outcome

Primary Study Outcome: Thromboembolic events—a composite of ischemic stroke, transient ischemic attack, stent thrombosis, urgent revascularization, and cerebrovascular mortality at 7 days—are the main result. The composite thromboembolic endpoint was prespecified because its components share a common pathophysiology of peri-procedural thrombosis. Despite varying clinical severity, these events represent a continuous spectrum of ischemic complications and are conventionally combined as a single endpoint in neurointerventional studies. Ischemic stroke was defined as a newly developed acute focal neurological deficit confirmed by clinical or imaging evidence of cerebral infarction, with non-ischemic causes ruled out; or a rapid worsening of a pre-existing focal neurological deficit determined to be caused by a new infarct (21). TIA was described as a brief period of neurological impairment brought on by focal cerebral ischemia that did not show signs of a permanent cerebral infarction on follow-up imaging (22). A cerebral infarction associated with the stented segment that was verified by angiography was referred to as stent thrombosis. Following stent implantation, cerebrovascular (any vessel) revascularization is determined within 24 h following a repeat episode of cerebral infarction. Death resulting from a known cerebrovascular etiology is referred to as cerebrovascular death. Secondary research findings: The composite of thromboembolic events that occurred within 30 days following the surgery served as the secondary outcome. According to the Thrombolysis in Myocardial Infarction (TIMI) classification (23) of hemorrhagic events, the safety outcome is a composite of major, mild, or minimal bleeding events within 30 days following the surgery. Fatal or intracranial bleeding, as well as any other bleeding that compromises hemodynamics and necessitates care, are considered significant bleeding. Any overt bleeding event that did not fit the major and minor criteria was considered minimal bleeding. A minor bleeding event is defined as bleeding that requires medical intervention. Three members of the Clinical Events Committee, who were blind to the treatment group assignment, independently evaluated all reported composite clinical thromboembolic events and safety outcomes. A fourth committee member decided any disputes. In order to make a final decision based on consensus, every controversial case was thoroughly reviewed by the entire five-member committee. No missing data were present for the variables included in the primary analyses, as the study only enrolled patients with complete clinical records (see exclusion criteria).

### Statistical methods

Statistical analyses were carried out with SPSS software (version 27.0). Categorical data were compared between the Monitored and Non-monitored Groups using the chi-squared test. Continuous variables are reported as mean ± standard deviation (x ± s) if normally distributed, with comparisons conducted using the independent samples t-test. If non-normally distributed, they are stated as median M (Q_25_, Q_75_) and compared using the Mann–Whitney U test. Cox regression models were used to analyze time-to-event outcomes, and the results were provided as *p* values, hazard ratios (HRs), and 95% confidence intervals (CIs). Kaplan–Meier (K-M) curves were plotted, and event-free cumulative incidence was calculated using the Kaplan–Meier method. To control for confounding due to the non-randomized allocation, propensity score matching (PSM) was first performed using a 1:1 nearest-neighbor algorithm with a caliper of 0.02, incorporating all baseline variables. Balance after matching was assessed by standardized mean differences (SMD), with SMD < 0.1 indicating good balance. Subsequently, a parsimonious multivariable Cox regression model was fitted in the original cohort, adjusting for center, CHA₂DS₂-VASc score, and aneurysm maximal diameter. The proportional hazards assumption for the Cox regression models was tested using Schoenfeld residuals. Center-level clustering was addressed by including center as a fixed covariate in all Cox models. Prespecified subgroup analyses for the primary and safety outcomes were conducted based on relevant clinical variables in the overall population; these analyses were exploratory in nature and were not repeated in the PSM cohort due to the limited number of events. A two-sided *p* value of less than 0.05 was considered statistically significant.

## Results

### Baseline characteristics

Analysis of patient baseline characteristics revealed no significant differences between the two groups for most variables, except for hematocrit (*p* = 0.012). After PSM, 115 pairs of patients were successfully matched. In the matched cohort, all baseline covariates were well balanced between the two groups, the majority of covariates achieved SMD < 0.1, with only a few variables showing SMD values between 0.1 and 0.2 (Table 1). All 368 patients completed the 30-day follow-up, with a median follow-up duration of 30 days (IQR, 30–30 days). No patients were lost to follow-up within the 30-day observation period.

**Table 1:** Comparison of general baseline data of the two groups of patients.

Characteristic	Before PSM				After PSM			
	Non-monitored group (*n* = 149)	Monitored group (*n* = 219)	*SMD*	*p*-value	Non-monitored group (*n* = 115)	Monitored group (*n* = 115)	*SMD*	*P*-value
Age [years, M (P_25_, P_75_)]	55.00 (49.00, 63.00)	56.00 (49.00, 61.00)	0.014	0.843	56.00 (50.00, 62.50)	55.00 (49.00, 61.00)	−0.060	0.432
BMI [kg/m^2^, M (P_25_, P_75_)]	25.00 (22.90, 27.23)	24.59 (22.26, 26.66)	−0.144	0.123	24.94 (23.22, 26.95)	24.73 (22.34, 26.950)	−0.000	0.925
Center (n, %)				0.903				1.000
A	69 (46.31)	100 (45.66)	−0.013		55.00 (47.83)	55.00 (47.83)	0.000	
B	80 (53.69)	119 (54.34)	0.013		60.00 (52.17)	60.00 (52.17)	0.000	
CHA₂DS₂-VASc	2.00 (1.00, 2.50)	2.00 (1.00, 2.00)	−0.032	0.826	2.00 (1.00, 2.00)	2.00 (1.00, 2.00)	0.000	0.948
Sex (n, %)				0.296				0.789
Male	59.00 (39.60)	100.00 (45.66)	0.122		49.00 (42.61)	47.00 (40.87)	−0.035	
Female	90.00 (60.40)	119.00 (55.09)	−0.122		66.00 (57.39)	68.00 (59.13)	0.035	
Symptom (n, %)	96.00 (64.43)	159.00 (72.60)	0.183	0.120	72.00 (62.61)	77.00 (66.96)	0.092	0.490
Pre-mRS [M (P_25_, P_75_)]	0.00 (0.00, 1.00)	0.00 (0.00, 1.00)	0.014	0.895	0.00 (0.00, 1.00)	0.00 (0.00, 1.00)	−0.071	0.596
Smoking (n, %)	38.00 (25.50)	64.00 (29.23)	0.082	0.507	29.00 (25.22)	34.00 (29.57)	0.095	0.460
Drinking (n, %)	20.00 (13.42)	43.00 (19.63)	0.156	0.158	17.00 (14.78)	22.00 (19.13)	0.111	0.772
Medical history								
Hypertension (n, %)	75.00 (50.34)	108.00 (49.32)	−0.020	0.931	56.00 (48.70)	58.00 (50.43)	0.035	0.792
Diabetes (n, %)	36.00 (24.16)	59.00 (26.94)	0.063	0.634	28.00 (24.35)	27.00 (23.48)	−0.021	0.877
Hyperlipidemia (n, %)	39.00 (26.17)	75.00 (34.25)	0.170	0.126	32.00 (27.83)	35.00 (30.43)	0.057	0.663
Prior cerebrovascular event								
Ischemic stroke (n, %)	23.00 (15.44)	41.00 (18.72)	0.084	0.499	21.00 (18.26)	21.00 (18.26)	0.000	1.000
Hemorrhagic stroke (n, %)	2.00 (1.34)	5.00 (2.28)	0.063	0.802	2.00 (1.74)	1.00 (0.87)	−0.094	1.000
Prior cardiovascular event								
Heart failure (n, %)	0.00 (0.00)	3.00 (1.37)	0.118	0.399	0.00 (0.00)	0.00 (0.00)	0.000	1.000
Coronary artery disease (n, %)	5.00 (3.36)	10.00 (4.57)	0.058	0.758	5.00 (4.35)	4.00 (3.48)	−0.047	1.000
Concomitant medication								
Insulin (n, %)	13.00 (8.72)	28.00 (12.79)	0.122	0.295	10.00 (8.70)	11.00 (9.57)	0.030	0.819
Metformin (n, %)	10.00 (6.71)	11.00 (5.02)	−0.077	0.648	4.00 (3.48)	6.00 (5.22)	0.078	0.518
ACE inhibitor (n, %)	5.00 (3.36)	15.00 (6.85)	0.138	0.224	5.00 (4.35)	6.00 (5.22)	0.039	0.757
Calcium channel blocker (n, %)	41.00 (27.52)	78.00 (35.62)	0.169	0.129	39.00 (33.91)	31.00 (26.96)	−0.157	0.252
Proton-pump inhibitor (n, %)	16.00 (10.74)	35.00 (15.99)	0.143	0.202	15.00 (13.04)	16.00 (13.91)	0.025	0.847
Statin (n, %)	33.00 (22.15)	56.00 (25.57)	0.078	0.529	31.00 (26.96)	27.00 (23.48)	−0.082	0.544
DAPT [d, M (P_25_, P_75_)]	7.00 (5.00, 8.00)	6.00 (5.00, 8.00)	0.033	0.825	7.00 (5.00, 8.00)	6.00 (5.00, 8.00)	−0.101	0.249
Hematological and coagulation indicators								
White blood cell count (per μL, $\bar{x}\pm s$ )	6.02 ± 1.73	6.34 ± 2.11	0.152	0.112	6.06 ± 1.80	6.17 ± 2.30	0.049	0.682
Red blood cell count (×10^6^/μL, $\bar{x}\pm s$ )	4.58 ± 0.52	4.47 ± 0.56	−0.182	0.078	4.53 ± 0.51	4.57 ± 0.52	0.075	0.565
Hemoglobin level (g/dL, $\bar{x}\pm s$ )	137.45 ± 15.08	138.03 ± 15.33	0.038	0.719	137.08 ± 15.19	139.73 ± 15.60	0.170	0.194
Hematocrit (%, $\bar{x}\pm s$ )	42.27 ± 4.15	41.08 ± 4.61	−0.258	**0.012**	42.24 ± 4.28	42.30 ± 4.20	0.015	0.913
Platelet count (×10^3^/μL, $\bar{x}\pm s$ )	239.93 ± 51.59	241.72 ± 53.13	0.034	0.749	242.08 ± 53	237.49 ± 47.21	−0.097	0.488
Pre-APTT (sec, $\bar{x}\pm s$ )	31.45 ± 3.86	32.01 ± 4.12	0.136	0.191	31.62 ± 3.78	31.94 ± 3.95	0.081	0.529
Aneurysm data								
Maximum size (mm, $\bar{x}\pm s$ )	6.08 ± 2.87	6.22 ± 2.57	0.055	0.624	6.12 ± 2.80	6.22 ± 2.67	0.041	0.765
Neck size (mm, $\bar{x}\pm s$ )	4.88 ±2.12	5.02 ± 2.99	0.050	0.597	4.84 ± 2.16	5.06 ± 2.97	0.073	0.527
Aspect ratio ( $\bar{x}\pm s$ )	1.18 ± 0.33	1.21 ± 0.38	0.080	0.424	1.17 ± 0.33	1.17 ± 0.38	−0.011	0.931
Location (n, %)				0.693				0.324
Anterior cerebral artery	27.00 (18.12)	30.00 (13.70)	−0.129		24.00 (20.87)	16.00 (13.91)	−0.201	
Internal carotid artery	88.00 (59.06)	135.00 (61.64)	0.053		62.00 (53.91)	75.00 (65.22)	0.237	
Middle cerebral artery	12.00 (8.05)	21.00 (9.59)	0.052		10.00 (8.70)	7.00 (6.09)	−0.109	
Posterior circulation	22.00 (14.77)	33.00 (15.07)	0.008		19.00 (16.52)	17.00 (14.78)	−0.049	
Procedural data								
Procedure time (min, $\bar{x}\pm s$ )	149.50 ± 32.50	154.50 ± 42.51	0.118	0.226	151.30 ± 34.62	149.80 ± 43.72	−0.034	0.774
Stent amount [n, M (P_25_, P_75_)]	1.00 (0.00, 1.00)	1.00 (0.00, 1.00)	-	1.000	1.00 (1.00, 1.00)	1.00 (1.00, 1.00)	0.000	-
Stent length [mm, M (P_25_, P_75_)]	14.00 (14.00, 22.00)	14.00 (14.00, 22.00)	0.182	0.079	14.00 (14.00, 22.00)	14.00 (14.00, 22.00)	0.091	0.484
Immediate occlusion grade (n, %)				0.279				0.626
Complete	118.00 (79.19)	186.00 (84.93)	0.160		92.00 (80.00)	96.00 (83.48)	0.094	
Residual neck	18.00 (12.08)	22.00 (10.05)	−0.068		16.00 (13.91)	15.00 (13.04)	−0.026	
Residual sac	13.00 (8.72)	11.00 (5.02)	−0.169		7.00 (6.09)	4.00 (3.48)	−0.142	

PSM, propensity score matching; BMI, Body mass index; ACE, angiotensin-converting enzyme; APTT, activated partial thromboplastin time; DAPT, dual antiplatelet therapy. Bold values indicate statistical significance (*p* < 0.05)..

### Primary clinical outcomes

In the original cohort, the primary thromboembolic outcome occurred in 7 of 219 patients (3.20%) in the Monitored Group and 12 of 149 patients (8.05%) in the Non-monitored Group (Table 2). In univariable Cox regression (Model 1), the monitored strategy was associated with a lower incidence of the primary outcome (HR = 0.388, 95% CI: 0.153–0.985, *p* = 0.045; Table 2). After adjusting for center, CHA₂DS₂-VASc score, and aneurysm maximal size (Model 2), the association remained consistent (adjusted HR = 0.391, 95% CI: 0.154–0.994, *p* = 0.048; Table 2). Stent thrombosis and TIA were rare, with no statistically significant changes between groups (*p* > 0.05). One cerebrovascular death occurred in the Non-monitored Group; no deaths from any cause occurred in the Monitored Group. Figure 2A displays the cumulative Kaplan–Meier event curves for the primary outcome, showing a lower cumulative event rate in the Monitored Group (3.20%) compared to the Non-monitored Group (8.05%; *p* < 0.05). In the PSM cohort (115 pairs), the event rate was 4.35% (5/115) in the Monitored Group and 8.70% (10/115) in the Non-monitored Group (HR = 0.487, 95% CI: 0.166–1.424, *p* = 0.189; Table 3), with the direction of association consistent with the main analysis. The proportional hazards assumption was tested using Schoenfeld residuals and was satisfied (global *p* > 0.05).

**Table 2:** Study outcomes in the monitored and non-monitored groups (n, %).

Outcomes	Non-monitored group (*n* = 149)	Monitored group (*n* = 219)	Model 1		Model 2	
			HR (95% CI)	*p*-value	HR (95% CI)	*p*-value
Primary thromboembolic outcomes	12.00 (8.05)	7.00 (3.20)	0.388 (0.153–0.985)	0.045	0.391 (0.154–0.994)	0.048
Ischemic stroke	9.00 (6.04)	5.00 (2.28)	0.370 (0.124–1.105)	0.075	0.374 (0.125–1.115)	0.078
Stent thrombosis	1.00 (0.67)	1.00 (0.46)	0.668 (0.042–10.681)	0.775	0.675 (0.042–10.802)	0.781
Transient ischemic attack	1.00 (0.67)	1.00 (0.46)	0.685 (0.043–10.951)	0.789	0.690 (0.043–11.046)	0.793
Death from cerebrovascular causes	1.00 (0.67)	0.00 (0.00)	-	-	-	-
Death from any cause	1.00 (0.67)	0.00 (0.00)	-	-	-	-
Secondary thromboembolic outcomes	16.00 (10.74)	10.00 (4.57)	0.412 (0.190–0.908)	0.026	0.412 (0.187–0.909)	0.028
Ischemic stroke	9.00 (6.04)	6.00 (2.74)	0.444 (0.158–1.248)	0.124	0.447 (0.159–1.256)	0.126
Stent thrombosis	2.00 (1.34)	2.00 (0.91)	0.667 (0.094–4.733)	0.685	0.666 (0.094–4.733)	0.685
Transient ischemic attack	2.00 (1.34)	1.00 (0.46)	0.340 (0.031–3.754)	0.379	0.343 (0.031–3.790)	0.383
Death from cerebrovascular causes	2.00 (1.34)	1.00 (0.46)	0.334 (0.030–3.686)	0.371	0.318 (0.029–3.506)	0.349
Death from any cause	1.00 (0.67)	0.00 (0.00)	-	-	-	-
Primary bleeding outcome: any bleeding	6.00 (4.03)	14.00 (6.39)	1.606 (0.617–4.178)	0.330	1.602 (0.616–4.170)	0.334
Major	1.00 (0.67)	1.00 (0.46)	0.681 (0.043–10.889)	0.786	0.688 (0.043–11.008)	0.792
Minor	1.00 (0.67)	3.00 (1.37)	2.043 (0.213–19.640)	0.536	2.061 (0.214–19.813)	0.531
Minimal	4.00 (2.68)	10.00 (4.57)	1.717 (0.539–5.476)	0.361	1.703 (0.534–5.429)	0.368

HR indicates hazard ratio; CI, confidence interval; Model 1, univariable Cox regression in the original cohort (*n* = 368), unadjusted.; Model 2, multivariable Cox regression in the original cohort (*n* = 368), adjusted for center; CHA_2_DS_2_-VASc score and Aneurysm maximum size.

**Figure 2:**
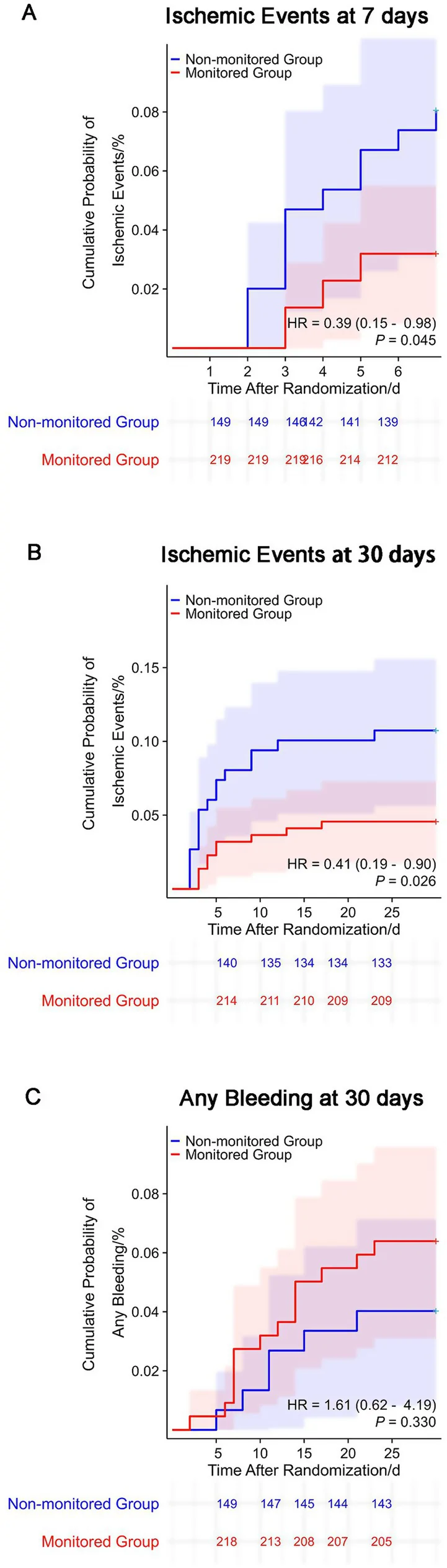
Cumulative incidence statistics. HR indicates hazard ratio. **(A)** Ischemic events at 7 days; **(B)** ischemic events at 30 days; **(C)** any bleeding at 30 days.

**Table 3:** Study outcomes in the monitored and non-monitored groups after PSM (n, %).

Outcomes	Non-monitored group (*n* = 115)	Monitored group (*n* = 115)	HR (95% CI)	*p*-value
Primary thromboembolic outcomes	10.00 (8.70)	5.00 (4.35)	0.487 (0.166–1.424)	0.189
Ischemic stroke	7.00 (6.09)	4.00 (3.48)	0.557 (0.163–1.902)	0.350
Stent thrombosis	1.00 (0.87)	0.00 (0.00)	-	0.610
Transient ischemic attack	1.00 (0.87)	1.00 (0.87)	0.991 (0.062–15.842)	0.995
Death from cerebrovascular causes	1.00 (0.87)	0.00 (0.00)	-	0.610
Death from any cause	0.00 (0.00)	0.00 (0.00)	-	-
Secondary thromboembolic outcomes	13.00 (11.30)	6.00 (5.22)	0.446 (0.169–1.172)	0.101
Ischemic stroke	7.00 (6.09)	4.00 (3.48)	0.557 (0.163–1.902)	0.350
Stent thrombosis	2.00 (1.74)	0.00 (0.00)	-	0.969
Transient ischemic attack	1.00 (0.87)	1.00 (0.87)	0.991 (0.062–15.842)	0.995
Death from cerebrovascular causes	2.00 (1.74)	1.00 (0.87)	0.497 (0.045–5.483)	0.568
Death from any cause	1.00 (0.87)	0.00 (0.00)	-	0.610
Primary bleeding outcome: any bleeding	5.00 (4.35)	10.00 (8.70)	2.049 (0.700–5.995)	0.190
Major	1.00 (0.87)	1.00 (0.87)	0.996 (0.062–15.921)	0.998
Minor	1.00 (0.87)	1.00 (0.87)	0.995 (0.062–15.915)	0.997
Minimal	3.00 (2.61)	8.00 (6.96)	2.714 (0.720–10.233)	0.140

HR indicates hazard ratio; CI, confidence interval; PSM, propensity score matching. Cox regression in the 1:1 PSM cohort (*n* = 115), adjusted for center.

### Secondary clinical outcomes

The overall incidence of the secondary thromboembolic outcome in the original cohort was 4.57% (10/219) in the Monitored Group compared to 10.74% (16/149) in the Non-monitored Group (Table 2). In univariable Cox regression (Model 1), the monitored strategy was associated with a lower incidence of the secondary outcome (HR = 0.412, 95% CI: 0.190–0.908, *p* = 0.026; Table 2). After adjusting for center, CHA₂DS₂-VASc score, and aneurysm maximal size (Model 2), the association remained consistent (adjusted HR = 0.412, 95% CI: 0.187–0.909, *p* = 0.028; Table 2). Nevertheless, there were no statistically significant differences between the groups in the secondary outcome’s individual component events (all *p* > 0.05; Table 2). Figure 2B cumulative Kaplan–Meier event curves for the secondary outcome suggest that the Monitored Group’s cumulative event rate was lower than that of the Non-monitored Group (*p* < 0.05). In the PSM cohort (115 pairs), the secondary outcome occurred in 5.22% (6/115) of the Monitored Group and 11.30% (13/115) of the Non-monitored Group (HR = 0.446, 95% CI: 0.169–1.172, *p* = 0.101; Table 3), with the direction of association consistent with the main analysis.

### Safety outcomes

In the original cohort, bleeding events within 30 days occurred in 14 out of 219 patients (6.39%) in the Monitored Group and 6 out of 149 patients (4.03%) in the Non-monitored Group (Table 2). In univariable Cox regression (Model 1), the monitored strategy was not associated with a significantly increased risk of any bleeding (HR = 1.606, 95% CI: 0.617–4.178, *p* = 0.330; Table 2). After adjusting for center, CHA₂DS₂-VASc score, and aneurysm maximal size (Model 2), the result remained consistent (adjusted HR = 1.602, 95% CI: 0.616–4.170, *p* = 0.334; Table 2). One patient in each group experienced significant bleeding, according to additional stratification (HR = 0.681, 95% CI: 0.043–10.889, *p* = 0.786; adjusted HR = 0.688, 95% CI: 0.043–11.008, *p* = 0.792). Three patients (1.37%) in the Monitored Group and one patient (0.67%) in the Non-monitored Group had minor bleeding (HR = 2.043, 95% CI: 0.213–19.640, *p* = 0.536; adjusted HR = 2.061, 95% CI: 0.214–19.813, *p* = 0.531). Ten patients (4.57%) in the Monitored Group and four patients (2.68%) in the Non-monitored Group experienced minimal bleeding events (HR = 1.717, 95% CI: 0.539–5.476, *p* = 0.361; adjusted HR = 1.703, 95% CI: 0.534–5.429, *p* = 0.368; Table 2). The Kaplan–Meier curve for any bleeding event within 30 days is presented in Figure 2C. In the PSM cohort (115 pairs), the monitored group showed a higher but non-significant rate of any bleeding (8.70% vs. 4.35%; HR = 2.049, 95% CI: 0.700–5.995, *p* = 0.190; Table 3), with consistent findings across major, minor, and minimal bleeding subtypes. The direction of association was consistent with the main analysis, although none of the comparisons reached statistical significance.

### Subgroup analysis of the outcome

Prespecified subgroup analyses conducted across the 368 enrolled patients showed no significant interaction association for either the primary or safety outcomes. Subgroups based on sex, age (≤65 years vs. >65 years), smoking status, drinking status, aneurysm size (≤7 mm vs. >7 mm), procedure time (≤2 h vs. >2 h), number of stents (1 vs. ≥2), history of prior ischemic stroke, and the presence of atherosclerotic lesions demonstrated hazard ratios consistent in direction with the overall treatment association. All 95% CIs for the subgroup HRs crossed 1.0, and all *p* values for interaction were greater than 0.05. This indicates that the observed association of the monitoring strategy with ischemic events was consistent across patients with different baseline characteristics, with no significant association modifiers identified (Figures 3, 4).

**Figure 3:**
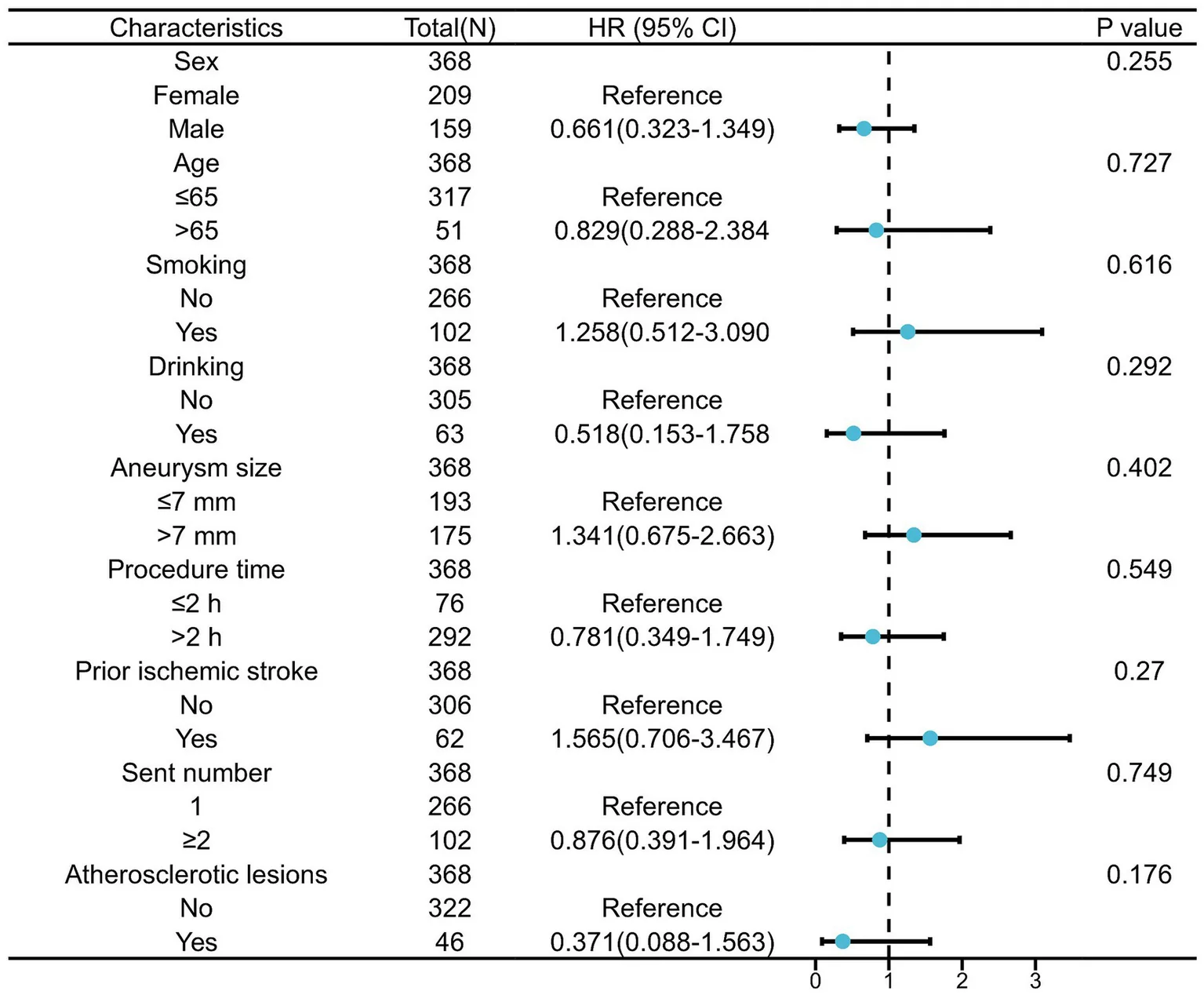
Subgroup analysis of the primary outcomes. HR indicates hazard ratio.

**Figure 4:**
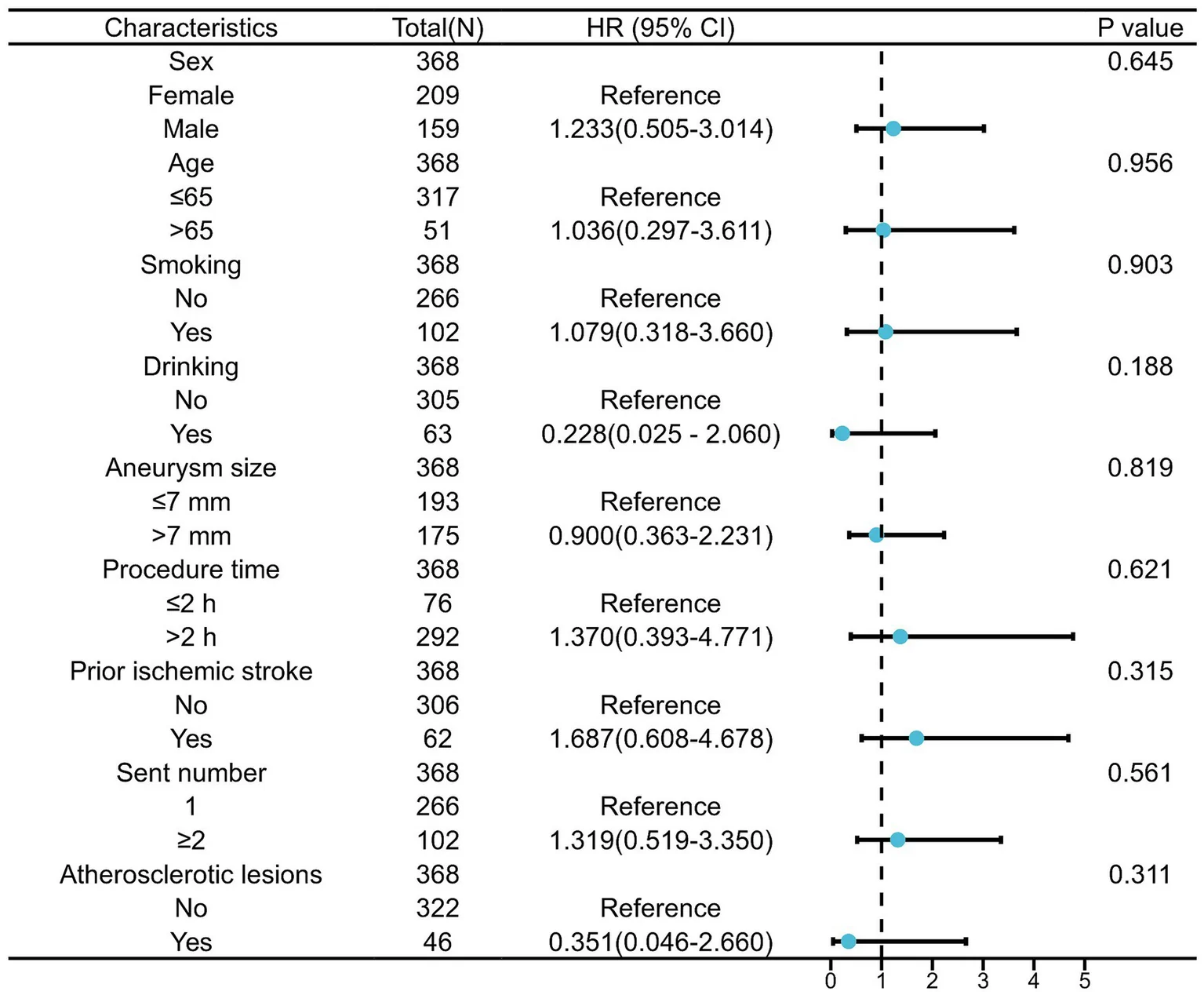
Subgroup analysis of safety outcomes. HR indicates hazard ratio.

## Discussion

In this retrospective study, we evaluated the association of a PFT-guided intensified antiplatelet strategy with peri-procedural thromboembolic events in UIA patients undergoing EP2 stent-assisted coiling. In the original cohort, the strategy was associated with a lower incidence of the primary composite outcome (adjusted HR = 0.391, 95%CI 0.154–0.994, *p* = 0.048). This association remained directionally consistent in propensity score-matched analysis (HR = 0.487, *p* = 0.189). These findings suggest that the comprehensive strategy may be associated with a reduced risk of early ischemic events, without a significant increase in major bleeding. It is important to emphasize that the observed association cannot be attributed solely to PFT itself, as the Monitored Group received a composite strategy including PFT screening, clopidogrel loading, aspirin dose escalation, and short-term tirofiban bridging in patients with HPR. Given the potent and rapid platelet inhibition provided by intravenous tirofiban, a substantial portion of the early observed association is likely driven by this bridging therapy. PFT enables identification of patients with inadequate platelet inhibition who may benefit from intensification, while avoiding unnecessary escalation in those with adequate response. Therefore, our findings should be interpreted as the net clinical association of the entire PFT-guided strategy, rather than as the association of PFT as a standalone test.

Our primary findings align with, yet also present noteworthy distinctions from, previous research on individualized antiplatelet therapy in neurointervention and broader cardiocerebrovascular fields. For instance, in patients with coronary artery disease undergoing Percutaneous Coronary Intervention (PCI), extensive studies have confirmed that HPR to clopidogrel is a strong predictor of stent thrombosis and major adverse cardiovascular events (23). For such patients, intensifying antiplatelet therapy guided by PFT or genotyping has been reported to be associated with a lower incidence of ischemic events without significantly increasing major bleeding risk (24, 35–37). Extending this concept to neurointervention, particularly intracranial stent placement, shares a logical foundation: ensuring adequate platelet inhibition is crucial for preventing device-related and procedure-related thrombosis. In the study by Zhou et al. (14), the Test group reported a 7-day stroke rate and 30-day major bleeding rate of 6.8 and 24%, respectively. In contrast, our Monitored Group had a lower 7-day event rate of 3.20% and a 30-day major bleeding rate of only 6.39%, suggesting that the immediate “loading dose + tirofiban bridge” strategy for clopidogrel HPR in a carefully selected aneurysm strategy cohort may be associated with more rapid platelet inhibition and a reduced high-thrombotic-risk window within the first 48 h post-procedure compared to switching P2Y12 inhibitors or increasing maintenance doses. Tirofiban, a rapid-onset, potent intravenous glycoprotein IIb/IIIa receptor inhibitor, provides immediate platelet inhibition during the high-thrombotic-risk peri-procedural period by completely blocking the final common pathway of platelet aggregation (25, 26). The underlying mechanisms remain speculative. One possible explanation may involve the specific characteristics of the EP2 stent—a self-expanding nitinol braided stent with higher metal coverage than open-cell designs, potentially delaying endothelialization. The exposed metal struts and coil mesh can create multiple cavity-like structures, potentially increasing local shear stress and promoting GP IIb/IIIa-mediated platelet activation (27). If a patient concurrently carries CYP2C19 loss-of-function alleles, leading to insufficient generation of active metabolites, standard 75 mg clopidogrel may inadequately occupy P2Y12 receptors, creating a fertile ground for thrombosis in this HPR state (28, 29). Our protocol, administering a 300 mg load and a 12-h tirofiban bridge within 24 h pre-procedure, essentially “shuts down” the platelet final common pathway when P2Y12 inhibition is insufficient, offering immediate protection during this most vulnerable period. This explanation aligns with findings from Yang et al. (30), whose meta-analysis suggested that combining tirofiban with conventional treatment in Acute Ischemic Stroke (AIS) was associated with improved functional outcomes. It is also corroborated by the earlier findings of Zi et al. (31), who reported significantly fewer thromboembolic events without increased bleeding in a tirofiban subgroup compared to a clopidogrel subgroup. Therefore, the observed association with early ischemic events may be partly related to the short-term application of tirofiban, potentially compensating for the delayed onset or inadequate response to oral P2Y12 inhibitors. Studies not showing such an association might lack such potent and timely rescue measures. Notably, the HR for ischemic stroke was 0.37 in our study, while stent thrombosis occurrences were rare. This suggests that EP2-related thrombotic events might manifest more as distal embolization rather than in-stent occlusion, consistent with a pathophysiological model where the stent’s larger cell size and struts potentially lead to platelet-leukocyte microemboli dislodging into distal perforators (32).

Our results provide further supportive evidence regarding secondary outcomes and safety. Although the individual event rates of the secondary 30-day thromboembolic outcome did not reach independent significance, the composite outcome HR of 0.41 still indicates a sustained beneficial trend favoring the Monitored Group. This might suggest that the curves converged over time, with the additional benefit of monitoring strategy concentrated predominantly in the very early phase. This “early effect attenuation” phenomenon has also been observed in studies by Zhou (14) and Li (33)et al. The reason may be that endothelial coverage of the stent begins after day 7, reducing metal exposure area and weakening platelet activation signals, thereby naturally narrowing the antithrombotic gap between PFT-guided intensified therapy and conventional treatment. Regarding safety, the lack of significant difference in bleeding events between groups aligns with the logic of the individualized PFT protocol: it intensifies therapy only for HPR patients, rather than universally increasing drug doses, and the tirofiban infusion was limited to 12 h, avoiding the bleeding risks associated with prolonged use. This contrasts with some prior PFT protocols where over-intensification of antiplatelet regimens led to increased bleeding, such as the study by Harris et al. (34), which suggested that more potent DAPT might increase bleeding risk without reducing major adverse cardiovascular events. By employing strict PFT thresholds and a short-course intensification regimen, our study balanced ischemic and hemorrhagic risks. Notably, major bleeding events were exceedingly rare in both groups, indicating a low inherent risk of severe intracranial bleeding post-strategy, further supporting the clinical safety of the PFT-guided approach.

This study has several limitations. First, its retrospective observational design is inherently susceptible to selection bias and unmeasured confounding. Although we performed propensity score matching and multivariable adjustment to control for measured confounders, residual confounding from unmeasured factors cannot be fully excluded. Second, the primary outcome had a limited number of events (*n* = 19), and the upper bound of the 95% confidence interval for the adjusted effect estimate (0.994) approached the null value, reflecting the imprecision of the estimate. This underscores that our findings should be interpreted with caution and considered hypothesis-generating rather than definitive. Third, the composite thromboembolic endpoint combined events with varying clinical severity and mechanisms. Although this composite was prespecified and is conventionally used in neurointerventional studies, its heterogeneity is acknowledged, and the results should be interpreted with this in mind. Fourth, the study was conducted solely in an Asian population; our findings should be validated in multicenter, more diverse populations. Finally, follow-up was limited to 30 days postoperatively, lacking data on longer-term outcomes associated with the individualized antiplatelet strategy.

In conclusion, this retrospective multicenter study suggests that a PFT-guided intensified antiplatelet strategy was associated with a lower incidence of early thromboembolic events without a significant increase in major bleeding in UIA patients treated with EP2 stent-assisted coiling. These findings support the potential utility of this comprehensive strategy, although the independent contribution of each component requires further investigation in larger prospective studies. Future research directions should include conducting large-scale, prospective, randomized controlled trials to provide higher-level evidence, further defining optimal monitoring criteria and strategy thresholds, and extending the follow-up period to assess long-term outcomes.

## Data Availability

The original contributions presented in the study are included in the article/supplementary material, further inquiries can be directed to the corresponding author.
